# Population-Based Characterization of Menstrual Migraine and Proposed Diagnostic Criteria

**DOI:** 10.1001/jamanetworkopen.2023.13235

**Published:** 2023-05-15

**Authors:** Mona Ameri Chalmer, Lisette J. A. Kogelman, Henrik Ullum, Erik Sørensen, Maria Didriksen, Susan Mikkelsen, Khoa Manh Dinh, Thorsten Brodersen, Kaspar R. Nielsen, Mie Topholm Bruun, Karina Banasik, Søren Brunak, Christian Erikstrup, Ole Birger Pedersen, Sisse Rye Ostrowski, Jes Olesen, Thomas Folkmann Hansen

**Affiliations:** 1 Department of Neurology, Danish Headache Center, Copenhagen University Hospital, Glostrup, Denmark; 2 Statens Serum Institut, Copenhagen, Denmark; 3 Department of Clinical Immunology, Centre of Diagnostic Investigation, Rigshospitalet, Copenhagen, Denmark; 4 Department of Clinical Immunology, Aarhus University Hospital, Aarhus, Denmark; 5 Department of Clinical Immunology, Zealand University Hospital, Køge, Denmark; 6 Department of Clinical Immunology, Aalborg University Hospital, Aalborg, Denmark; 7 Department of Clinical Immunology, Odense University Hospital, Odense, Denmark; 8 Novo Nordisk Foundation Center for Protein Research, University of Copenhagen, Copenhagen, Denmark; 9 Department of Clinical Medicine, University of Copenhagen, Copenhagen, Denmark

## Abstract

**Importance:**

There is a need for better recognition and more extensive research into menstrual migraine (MM) in the general population, and a revision of the diagnostic criteria for MM is warranted to move the field forward. Increased understanding of MM is crucial for improving clinical care, diagnosis, and therapy for MM.

**Objectives:**

To assess the clinical characteristics of MM, including severity and treatment response, and to propose new diagnostic criteria for pure MM and menstrually related migraine.

**Design, Setting, and Participants:**

This is a case-control study of Danish individuals with migraine. All individuals completed a 105-item validated diagnostic migraine questionnaire, sent via the Danish electronic mailing system (e-Boks) between May and August 2020, allowing diagnosis of pure MM and menstrually related migraine by the *International Classification of Headache Disorders, Third Edition* (*ICHD-3*). Data analysis was performed from September 2021 to November 2022.

**Exposure:**

Diagnosis of migraine.

**Main Outcomes and Measures:**

Clinical characteristics of women with MM and women with nonmenstrual migraine (non-MM) were compared using the *ICHD-3 *diagnostic criteria. A simulation of the risk of randomly misclassifying MM was based on number of migraine attacks during 3 menstrual cycles (3 × 28 days), and simulation analyses were performed using 100 000 permutations of random migraine attacks in migraine patients.

**Results:**

A total of 12 618 individuals, including 9184 women, with migraine participated in the study. Among the women with migraine, the prevalence of MM was 16.6% (1532 women), and the prevalence of non-MM was 45.9% (4216 women). The mean (SD) age was 38.7 (8.7) years for women with MM and 37.0 (9.2) years for women with non-MM. Of the 1532 women with MM, 410 (26.8%) fulfilled *ICHD-3* diagnostic criteria for pure MM, 1037 (67.7%) fulfilled *ICHD-3* diagnostic criteria for menstrually related migraine, and 152 (9.9%) fulfilled proposed diagnostic criteria for rare pure MM. MM was associated with a higher frequency of migraine-accompanying symptoms (odds ratio [OR], 1.98; 95% CI, 1.71-2.29), more frequent (OR, 7.21; 95% CI, 5.77-9.03) and more severe (OR, 1.17; 95% CI, 1.13-1.21) migraine attacks, lower frequency of nonmigraine headache (OR, 0.31; 95% CI, 0.18-0.49), an overall greater response to treatment with triptans (OR, 1.66; 95% CI, 1.24-2.24), better improvement of migraine attacks during late pregnancy (OR, 5.10; 95% CI, 2.17-14.00), and faster reappearance of migraine attacks post partum (OR, 3.19; 95% CI, 2.40-4.25). Hormonal contraceptive–related MM was associated with a higher prevalence of migraine without aura than migraine related to spontaneous menstruation (OR, 1.82; 95% CI, 1.62-2.06). Otherwise, no differences between hormonal and spontaneous MM were observed. The risk of random diagnostic misclassification of *ICHD-3* menstrually related migraine in women with high frequency episodic migraine was 43%. This risk was reduced to 3% when applying the proposed criteria for menstrually related migraine.

**Conclusions and Relevance:**

In this case-control study, MM in the general population had clinical characteristics that were quantitively different from those of non-MM. Detailed descriptive data and suggested improved diagnostic criteria for pure MM and menstrually related migraine were provided.

## Key Points

**Question:**

Are the current *International Classification of Headache Disorders, Third Edition*, diagnostic criteria for menstrual migraine adequate?

**Findings:**

In this case-control study of 12 618 Danish individuals with migraine, menstrual migraine was associated with significantly more severe migraine attacks but was not adequately captured by the current diagnostic criteria.

**Meaning:**

Menstrual migraine is an important diagnostic entity, and these findings suggest that new diagnostic criteria are necessary.

## Teaser

This case-control study assesses the clinical characteristics of menstrual migraine and proposes new diagnostic criteria for menstrual migraine and menstrually related migraine.

## Introduction

Migraine is the second leading cause of years lived with disability globally among all ages, affecting women 2 to 3 times more often than men.^1,2^ Among women of childbearing age, migraine is the number 1 cause of lost years of healthy life, globally.^2^ In women, migraine varies enormously during puberty, the menstrual cycle, pregnancy, the post partum period, and menopause. Remarkably, 18% to 25% of women with migraine^3,4,5^ have migraine attacks associated with menstruation (ie, menstrual migraine [MM]). In women with MM, migraine attacks are associated with considerable disability.

The current diagnostic criteria in the *International Classification of Headache Disorders, Third Edition (ICHD-3)*^6^ for MM have critical issues that need to be addressed. Some of these issues have been discussed in other studies,^7,8,9,10^ and we further address these critical issues in our research. The first issue is that the criterion—migraine must occur in 2 of 3 consecutive menstrual cycles—does not consider migraine frequency, and the sensitivity and specificity of this criterion is debated.^9,10^ Diagnostic misclassification of menstrually related migraine may occur in women with chronic migraine (CM) or high-frequency episodic migraine (HFEM),^11^ since most women with 8 or more migraine days per month, just by chance, must have attacks within the 5-day perimenstrual window. The second issue is that women with rare migraine attacks occurring exclusively at menstruation (ie, rare pure MM) do not fulfill the criteria because the rare migraine attack frequency does not reach 2 of 3 consecutive menstrual cycles, but the association between migraine and menstruation is nevertheless absolute. The third issue of the current criteria is the timing of migraine attacks, since it is currently unclear what is meant by *occur* in the current criteria. Does *occur* mean that the migraine attacks begin and/or end on day 1 ± 2 of menstruation? The fourth issue is that it remains unclear whether women can have 2 separate attacks within day 1 ± 2 of menstruation. The aims of the present study were (1) to compare the clinical characteristics of women with MM with the clinical characteristics of women with nonmenstrual migraine (non-MM), (2) to analyze the 4 main issues of the current diagnostic criteria in a large population-based setting, and finally (3) to propose new appendix criteria for MM.

## Methods

### Study Design

This case-control study uses data from a population-based cohort of individuals with migraine from the Danish Migraine Population Cohort (DaMP), a subgroup of the Danish Blood Donor Study (DBDS),^12^ which is largely representative of the general Danish population with migraine. Written informed consent was obtained from all participants. The DBDS study was approved by the Regional Committees on Health Research Ethics and the Regional Data Protection Agency. This study followed the Strengthening the Reporting of Observational Studies in Epidemiology (STROBE) reporting guideline for case-control studies.

All participants completed a 105-item, in-cohort validated diagnostic headache questionnaire with a positive predictive value of 97% (eAppendix 1 in Supplement 1). Within the 105-item headache questionnaire, patients completed a 12-item physical health component scale (PCS-12), which assessed bodily pain, physical functioning, general health, and vitality; a 12-item self-perceived mental health scale (MCS-12), which assessed general health, vitality, mental health, emotional functioning, and social functioning; and a visual analog scale (VAS), which measured the intensity of pain of migraine attacks. The questionnaire also contained questions regarding treatment outcomes for triptans and over-the-counter analgesics. Treatment outcomes were scaled from 0 to 10 and defined as the interval from 50% pain relief to pain freedom. Menstruation-related questions in the diagnostic headache questionnaire included the following:

1. “Do you have migraine attacks in relation to your menstruation? That is, that the migraine attacks occur in the period from two days before the bleeding starts to two days after the day the bleeding has begun.”
2. “Do you have migraine attacks in connection with your menstruation in at least two out of three (2/3) of your menstrual cycles? That is, that the migraine attacks occur in the period of two days before the bleeding starts to two days after the day the bleeding has begun”.
3. “Do you exclusively have migraine attacks in relation to your menstruation? That is, that the migraine attacks occur in the period of two days before the bleeding starts to two days after the day the bleeding has begun and at no other time.”

In the analysis, pure MM and menstrually related migraine were defined by the *ICHD-3* criteria, and rare pure MM was defined by this study as rare migraine attacks that are exclusively related to menstruation.^6^ The group of women who had either *ICHD-3* pure MM, rare pure MM, or *ICHD-3* menstrually related migraine were collectively referred to as the MM group, and the group of women with migraine but not MM were collectively referred to as the non-MM group. Of the individuals from the DaMP cohort, men, women who had reached menopause, nonresponders of the MM questions on the diagnostic headache questionnaire and women not fulfilling *ICHD-3* MM diagnostic criteria were excluded from the analysis (eFigure in Supplement 1). Women with CM or HFEM were excluded from the MM group, and were included in the non-MM group.

### Statistical Analysis

Data analysis was performed from September 2021 to November 2022. Analyses were performed using R statistical software version 4.0.0 and R Studio statistical software version 1.3.1073 (both from R Project for Statistical Computing). Descriptive statistics and logistic regression analyses were used to assess the features of MM. Categorical variables were described as number (percentage) of participants. Results of number of participants between 0 to 4 were indicated as less than 5. A 2-tailed *P* < .05 denoted statistical significance. A simulation of the risk of randomly detecting MM was based on number of migraine attacks and calculated as a function of number of migraine attacks during 3 menstrual cycles (3 × 28 days). Analysis was done using 100 000 permutations of random migraine attacks in patients. The simulated MM function was used to generate the distribution of the test statistic under the null hypothesis of no difference between the MM and non-MM groups. This distribution was then used to calculate the *P* value and determine the statistical significance of the differences between the 2 groups. The simulated MM function allowed for the modeling of the following parameters: days with menstruation, length of the menstruation period in days, number of menstruation periods to simulate, number of migraine attacks, and number of patients simulated (eAppendix 2 in Supplement 1).

## Results

### Cohort Characteristics

The DaMP cohort consisted of 12 618 individuals (3434 men and 9184 women) with migraine; 5748 women answered the menstruation-related questions on the validated diagnostic migraine questionnaire. The mean (SD) age at interview was 38.7 (8.7) years for women with MM and 37.0 (9.2) years for women with non-MM. The prevalence of MM among all women with migraine in the DaMP cohort was 16.6% (1532 of 9184 women), and the prevalence of non-MM was 45.9% (4216 women). Among the 1532 women with MM, 410 (26.8%) fulfilled *ICHD-3* criteria for pure MM, 1037 (67.7%) fulfilled *ICHD-3* criteria for menstrually related migraine, and 152 (9.9%) fulfilled the study-defined criteria for rare pure MM. Among the 1037 women with menstrually related migraine, 67 (6.4%) had HFEM. They were excluded from the MM group and included in the non-MM group (eFigure in Supplement 1). Of the 1532 women with MM, 110 women (7.2%) had MM with aura attacks, 949 women (61.9%) had MM without aura attacks, and 473 women (30.9%) had MM with and without aura attacks (Table 1). Women in the MM group had a lower self-perceived physical health score measured on the PCS-12 than women in the non-MM group, (mean [SD] score, 54.7 [5.33] vs 55.2 [5.36]; odds ratio [OR], 0.98; 95% CI, 0.97-0.99; *P* < .001).

**Table 1.** Comparison of Clinical Characteristics of Women With Menstrual Migraine and Nonmenstrual Migraine^a^

Characteristic	Women, No. (%)		OR (95% CI)	*P* value
	Menstrual migraine (n = 1532)	Nonmenstrual migraine (n = 4216)		
Migraine subtypes				
Migraine with aura				
Any	110 (7.2)	881 (20.9)	0.29 (0.23-0.35)	<.001
Visual aura	107 (97.3)	857 (97.4)	0.93 (0.32-3.96)	.90
Sensory aura	15 (13.6)	176 (20.0)	0.63 (0.34-1.08)	.11
Speech and/or language aura	20 (18.3)	119 (13.6)	1.42 (0.82-2.35)	.19
Motor aura	14 (12.7)	38 (4.3)	3.35 (1.69-6.31)	.003
Migraine with and without aura	473 (30.9)	1355 (32.1)	0.96 (0.85-1.09)	.55
Migraine without aura	949 (61.9)	1980 (47.0)	1.82 (1.62-2.06)	<.001
Migraine attack frequency				
No migraine attacks the last 3 months	384 (25.1)	2329 (55.2)	1 [Reference]	NA
1-3 d/mo	930 (60.7)	1573 (37.3)	3.68 (3.21-4.22)	<.001
4-7 d/mo	218 (14.2)	190 (4.5)	7.21 (5.77-9.03)	<.001
≥8 d/mo^b^	0	124 (2.9)	NA	NA
Nonmigraine headache frequency				
Never	88 (5.8)	210 (5.0)	1 [Reference]	NA
<1 d/y	465 (30.6)	1114 (26.6)	1.03 (0.78-1.36)	.85
≥1 d/y	877 (57.7)	2069 (49.5)	1.00 (0.78-1.31)	.96
≥1 d/mo	64 (4.2)	605 (14.5)	0.24 (0.17-0.35)	<.001
≥1 d/wk	25 (1.6)	183 (4.4)	0.31 (0.18-0.49)	<.001
Migraine attack duration				
<4 h^c^	66 (4.3)	663 (15.8)	0.31 (0.23-0.40)	<.001
4-24 h	866 (56.6)	2723 (65.1)	1 [Reference]	NA
25-72 h	588 (38.4)	773 (18.5)	2.32 (2.03-2.65)	<.001
>72 h^c^	10 (0.65)	25 (0.6)	1.20 (0.55-2.45)	.62
Pain characteristics				
Unilateral pain	711 (46.6)	1550 (37.6)	1.45 (1.28-1.63)	<.001
Pulsatile pain	1361 (89.1)	3552 (85.1)	1.49 (1.24-1.79)	<.001
Routine activities exacerbate pain	1264 (82.9)	3240 (78.3)	1.40 (1.20-1.63)	<.001
Accompanying symptoms				
Nausea	1255 (82.1)	2933 (70.1)	1.98 (1.71-2.29)	<.001
Vomiting	799 (52.3)	1910 (45.7)	1.28 (1.14-1.44)	<.001
Photophobia	1405 (91.9)	3788 (90.3)	1.26 (1.02-1.56)	<.001
Phonophobia	1317 (86.2)	3346 (79.8)	1.60 (1.36-1.89)	<.001
Photophobia and phonophobia	1251 (81.9)	3199 (76.3)	1.43 (1.24-1.66)	<.001
Osmophobia	622 (40.8)	1354 (32.4)	1.41 (1.25-1.59)	<.001
Allodynia	421 (27.8)	827 (19.9)	1.56 (1.36-1.79)	<.001
Cranial autonomic symptoms^d^	600 (41.1)	1100 (27.4)	1.91 (1.68-2.16)	<.001
Positive outcomes of antimigraine treatment				
Triptans	365 (82.4)	559 (71.8)	1.66 (1.24-2.24)	<.001
Over-the-counter simple analgesics	714 (53.4)	1894 (52.7)	1.03 (0.91-1.17)	.66

Abbreviations: NA, not applicable; OR, odds ratio.

^a^ Women with nonmenstrual migraine (ie, without relation to menstruation) were used as reference in the adjusted logistic regression analysis (adjusted for age).

^b^ Women with high-frequency episodic migraine were excluded from the menstrual migraine group and were included in nonmenstrual migraine group.

^c^ Only includes participants who had migraine with aura.

^d^ Defined by the proposed diagnostic criteria for migraine with cranial autonomic symptoms.^13^

### Association of MM With Migraine Attack Frequency and Severity

The MM group had a higher frequency of migraine attacks (OR, 7.21; 95% CI, 5.77-9.03; *P* < *.*001) but a lower frequency of nonmigraine headaches (OR, 0.31; 95% CI, 0.18-0.49; *P* < .001) than the non-MM group (Table 1). The duration of migraine attacks was more likely to be longer for the MM group (OR, 2.32; 95% CI, 2.03-2.65; *P* < .001). The intensity of pain during migraine attacks, measured by the VAS, was higher for the MM group than for the non-MM group (mean [SD] score, 7.87 [1.48] vs 7.43 [1.87]; OR, 1.17; 95% CI, 1.13-1.21; *P* < .001). MM was also associated with more unilateral pain (OR, 1.45; 95% CI, 1.28-1.63; *P* < *.*001), pulsatile pain (OR, 1.49; 95% CI, 1.24-1.79; *P* < *.*001), and pain exacerbated by physical activity during attack (OR, 1.40; 95% CI, 1.20-1.63; *P* < *.*001). The associated symptoms nausea, vomiting, photophobia, phonophobia, osmophobia, allodynia and cranial autonomic symptoms^13^ were also more frequent in the MM group (OR, 1.98; 95% CI, 1.17-2.29; *P* < .001) than the non-MM group.

### Association of MM With Treatment Outcomes

As shown in Table 1, a total of 365 women in the MM group (82.4%) reported positive outcomes after treatment with triptans, whereas 559 women in the non-MM group (71.8%) reported positive outcomes after triptan treatment, suggesting that MM was associated with an overall greater response to treatment with triptans (OR, 1.66; 95% CI, 1.24-2.24; *P* < .001). There was no difference in the treatment outcomes of over-the-counter simple analgesics between the MM group and the non-MM group (714 women [53.4%] vs 1894 women [52.7%]; OR, 1.03; 95% CI, 0.91–1.17; *P* = .66).

Women in the MM group were more likely than women in the non-MM group to have tried a prophylactic drug (121 women [12.5%] vs 309 women [7.3%]; OR, 1.77; 95% CI, 1.46-2.14; *P* < *.*001). Among the prophylactic treatments assessed, women with MM had tried calcium channel blockers and hormone therapy significantly more often than women with non-MM (eTable 1 in Supplement 1). At time of enrollment, 60 women with MM (3.9%) were active users of prophylactic drugs, whereas 87 women with non-MM (2.1%) were active users of prophylactic drugs. Efficacy of prophylactic treatment was defined as at least 50% reduction in the frequency of days with migraine. Among active users, proportionally fewer women in the MM group reported efficacy of any prophylactic treatment than women in the non-MM group (40 women [69.5%] vs 64 women [75.3%]; OR, 0.71; 95% CI, 0.33-1.54; *P* = .39).

### Association of MM With Migraine Attacks During Late Pregnancy and Post Partum

Women with MM were more likely to have children than women with non-MM (1021 women [66.6%] vs 2485 women [58.9%]; OR, 1.18; 95% CI, 1.01-1.37; *P* = .03). There was no difference between the 2 groups in the total number of children per woman. There was no difference between women in the MM group (633 women [62.0%]) and women in the non-MM group (1533 women [61.7%]) regarding the prevalence of prepregnancy migraine (OR, 1.03; 95% CI, 0.89-1.21; *P* = .68). Women in the MM group were more likely to experience improvement of migraine attacks (OR, 5.10; 95% CI, 2.17-14.00; *P* < *.*001) or disappearance of migraine attacks (OR, 5.25; 95% CI, 2.30-14.20; *P* < *.*001) during the second and third trimester of pregnancy, than women in the non-MM group; however, women in the MM group were more likely to have migraine attacks reappear faster (ie, within 1 month) post partum (OR, 3.19; 95% CI, 2.40-4.25; *P* < .001) and less likely to have migraine attacks totally disappear post partum than women in the non-MM group (OR, 0.46; 95% CI, 0.24-0.85; *P* = .01) (eTable 2 in Supplement 1).

### Hormonal Contraceptive–Related MM vs Spontaneous MM

At time of the interview, proportionally more women in the MM group used hormonal contraceptives than women in the non-MM group (298 women [28.1%] vs 761 women [18.1%]). Women in the MM group using hormonal contraceptives were referred to as women with hormonal contraceptive–related MM. Women with hormonal contraceptive–related MM predominately had migraine without aura (OR, 1.82; 95% CI, 1.62-2.06) (Table 2). There was no difference in self-perceived physical health (mean [SD] PCS-12 score, 55.4 [4.7] vs 54.6 [5.5]; OR, 1.01; 95% CI, 0.98-1.03; *P* = .62) and self-perceived mental health (mean [SD] MCS-12 score, 50.1 [8.4] vs 51.4 [7.9]; OR, 1.00; 95% CI, 0.99-1.02; *P* = .86), between women with hormonal contraceptive–related MM and spontaneous MM. There was no difference in the intensity of pain during migraine attacks, measured by the VAS, between women with hormonal contraceptive–related MM and women with spontaneous MM (mean [SD] VAS score, 7.77 [1.40] vs 7.90 [1.50]; OR, 0.95; 95% CI, 0.87-1.04; *P* = .24).

**Table 2.** Comparison of Clinical Characteristics of Women With Hormonal Contraceptive–Related Menstrual Migraine and Spontaneous Menstrual Migraine^a^

Characteristic	Women, No. (%)		OR (95% CI)	*P* value
	Hormonal contraceptive–related menstrual migraine (n = 298)	Spontaneous menstrual migraine (n = 1233)		
Migraine subtypes				
Migraine with aura				
Any	110 (7.2)	881 (20.9)	0.29 (0.23-0.35)	<.001
Visual aura	127 (43.1)	490 (39.8)	1.05 (0.80-1.39)	.07
Sensory aura	39 (13.1)	167 (13.6)	0.76 (0.50-1.12)	.18
Speech and/or language aura	29 (9.8)	169 (13.7)	0.61 (0.39-0.94)	.03
Motor aura	27 (9.1)	107 (8.7)	0.74 (0.45-1.16)	.20
Migraine with and without aura	473 (30.9)	1356 (32.2)	0.96 (0.85-1.09)	.55
Migraine without aura	949 (61.9)	1979 (46.9)	1.82 (1.62-2.06)	<.001
Migraine attack frequency				
No migraine attacks the last 3 months	78 (26.2)	306 (24.8)	1 [Reference]	NA
1-3 d/mo	179 (60.1)	751 (60.9)	0.91 (0.66-1.25)	.56
4-7 d/mo	41 (13.8)	176 (14.3)	0.83 (0.53-1.30)	.43
Nonmigraine headache frequency				
Never	17 (5.7)	71 (5.8)	1 [Reference]	NA
<1 d/y	102 (34.3)	363 (29.7)	1.17 (0.65-2.22)	.61
≥1 d/y	161 (54.2)	716 (58.6)	1.21 (0.68-2.25)	.54
≥1 d/mo	13 (4.4)	50 (4.1)	1.32 (0.55-3.13)	.53
≥1 d/wk	4 (1.4)	21 (1.7)	1.20 (0.30-3.98)	.77
Migraine attack duration				
<4 h^b^	12 (4.0)	54 (4.4)	0.69 (0.33-1.34)	.29
4-24 h	190 (63.8)	676 (54.9)	1 [Reference]	NA
25-72 h	95 (31.9)	492 (40.0)	0.89 (0.67-1.20)	.45
>72 h^b^	1 (0.34)	9 (0.73)	0.69 (0.89-0.92)	.73
Pain characteristics				
Unilateral pain	136 (45.6)	575 (46.8)	1.03 (0.79-1.35)	.82
Pulsatile pain	279 (93.6)	1081 (88.0)	1.58 (0.97-2.72)	.08
Routine activities exacerbate pain	245 (82.5)	1018 (83.0)	0.76 (0.53-1.10)	.13
Accompanying symptoms				
Nausea	231 (77.5)	1023 (83.2)	1.03 (0.79-1.35)	.82
Vomiting	145 (48.7)	653 (53.1)	0.91 (0.70-1.20)	.52
Photophobia	271 (90.9)	1133 (92.1)	0.65 (0.41-1.07)	.08
Phonophobia	255 (85.9)	1061 (86.3)	0.82 (0.56-1.22)	.31
Photophobia and phonophobia	242 (81.5)	1008 (82.0)	0.81 (0.57-1.15)	.23
Osmophobia	90 (30.4)	531 (43.3)	0.58 (0.44-0.78)	<.001
Allodynia	73 (24.7)	347 (28.5)	0.75 (0.55-1.02)	.07
Cranial autonomic symptoms^c^	120 (42.3)	479 (40.8)	0.87 (0.65-1.15)	.33
Positive outcomes of antimigraine treatment				
Triptans	50 (79.4)	315 (82.9)	0.97 (0.48-2.06)	.93
Over-the-counter simple analgesics	143 (52.8)	570 (53.5)	0.90 (0.67-1.19)	.45

Abbreviations: NA, not applicable; OR, odds ratio.

^a^ Women with spontaneous menstrual migraine were used as reference in the adjusted logistic regression analysis (adjusted for age).

^b^ Only includes participants who had migraine with aura.

^c^ Defined by the proposed diagnostic criteria of migraine with cranial autonomic symptoms.^13^

### Proposed Diagnostic Criteria

On the basis of our data and the literature, we propose new diagnostic criteria for MM. Our data show that rare pure MM is not uncommon in women who do not fulfill current diagnostic criteria because they do not have attacks in at least 2 of 3 consecutive menstrual cycles. The literature has already suggested that HFEM and CM should be exempt from the MM diagnosis,^10^ and our data suggest a lower frequency value of migraine attacks. For the classification, menstruation is defined as an endometrial bleeding resulting either from the normal menstrual cycle or from the withdrawal of exogenous progestogens as in the use of oral contraceptives or cyclical hormone replacement therapy. The first day of menstruation is day 1, and the preceding day is day −1; there is no day 0. We propose diagnostic appendix criteria for pure MM (Box 1) and menstrually related migraine (Box 2).

Box 1. **Proposed Diagnostic Appendix Criteria for A1.1 Pure Menstrual Migraine**

- Attacks in menstruating women fulfilling the diagnostic criteria for 1.1 migraine without aura and/or 1.2 migraine with aura
- Attacks occur exclusively in association with menstruation, beginning on day 1 ± 2 (ie, days −2 to +3) of menstruation and at no other times of the cycle^a,b^

^a^
Attacks during at least 3 menstruations are reported.

^b^
If after a menstrual migraine attack the patient is headache free for at least 24 hours, spontaneously or as a result of treatment, and migraine reoccurs within the −2 to +3-day menstrual interval, the attacks are regarded as separate menstrual migraine attacks.

Box 2. **Proposed Diagnostic Appendix Criteria for A2.1 Menstrually Related Migraine**

- Attacks in menstruating women fulfilling the diagnostic criteria for 1.1 migraine without aura and/or 1.2 migraine with aura
- At least half of all migraine attacks begin on day 1 ± 2 (ie, days −2 to +3) of menstruation^a,b^

^a^
Attacks during at least 3 menstruations are reported.

^b^
If after a menstrual migraine attack the patient is headache free for at least 24 hours, spontaneously or as a result of treatment, and migraine reoccurs within the −2 to +3-day menstrual interval, the attacks are regarded as separate menstrual attacks.

### Proposed Criteria for MM vs *ICHD-3* Criteria

The risk of diagnostic misclassification of the *ICHD-3* menstrually related migraine increased steeply in women who had 3 or more monthly migraine attacks (Figure). In women with HFEM, the risk of misclassification of *ICHD-3* menstrually related migraine was 43%. This risk was reduced to 3% when applying the proposed criteria for menstrually related migraine (eTables 3 and 4 in Supplement 1). The risk of misclassification of pure MM in women with rare pure MM was 17%. For a woman with 3 or more attacks per month, the *ICHD-3* pure MM criteria and the proposed criteria for pure MM performed equally well, with a misclassification rate of 0.5%, but the proposed criteria were inclusive of rare pure MM (Figure).

**Figure.**
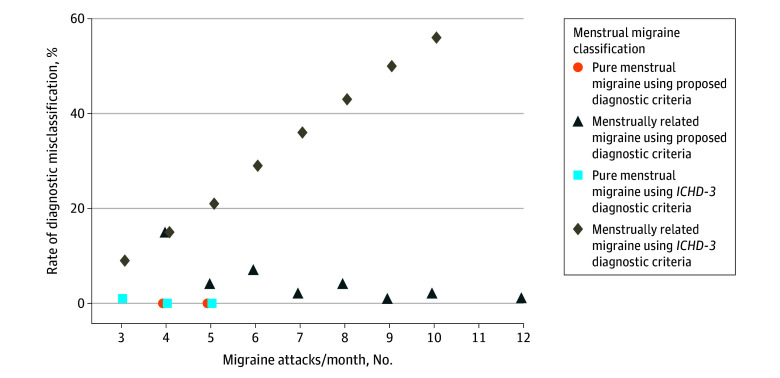
Rate of Diagnostic Misclassification of Menstrual Migraine Diagnoses Simulations were performed for a period of 3 menstrual cycles for proposed diagnostic criteria for pure menstrual migraine, proposed diagnostic criteria for menstrually-related migraine, *International Classification of Headache Disorders, Third Edition (ICHD-3) *diagnostic criteria for pure menstrual migraine, and *ICHD-3 *diagnostic criteria for menstrually related migraine. The figure shows the likelihood of diagnostic misclassification of menstrual migraine as a function of the number of migraine attacks. The analysis was done using 100 000 permutations of random attacks in migraine patients. The risk of diagnostic misclassification of menstrually related migraine was 43%, when applying the *ICHD-3* diagnostic criteria vs 3% when applying the proposed criteria for menstrually related migraine in women with 8 or more monthly migraine attacks in a period of 3 months (ie, high frequency episodic migraine and chronic migraine).

## Discussion

In this case-control study, we reported a prevalence of MM of 16.6% among menstruating women with migraine, which was similar to previously published population-based studies.^3,4,5,14^ We reported that among women with MM in a general population, 26.8% fulfilled *ICHD-3* diagnostic criteria for pure MM, 67.7% fulfilled *ICHD-3* diagnostic criteria for menstrually related migraine, and 9.9% fulfilled study-defined diagnostic criteria for rare pure MM. We also showed that women with MM in the general population had clinical characteristics that were quantitively different from those of women with non-MM. On the basis of our data, we proposed new diagnostic criteria for pure MM and menstrually related migraine.

### Clinical Characteristics of MM

In a comprehensive review, Vetvik et al^7^ noted that there is a need for better recognition and more extensive research into MM. Existing data on headache occurrence and increased intensity during important phases of the menstrual cycle are mostly based on clinical cohorts.

Our data showed that women with MM had a higher frequency of migraine-accompanying symptoms, more frequent and severe migraine attacks, lower frequency of nonmigraine headache, and better outcomes of treatment with triptans than women with non-MM. Our study is so far, to our knowledge, the largest population-based study with the primary aim to clinically characterize women with MM and compare them with women with non-MM. Previous population-based studies have not been large enough to properly assess the clinical characteristics of MM, and research has been hampered by an absence of clear and valid diagnostic criteria (eAppendix 3 in Supplement 1). Thus far, 1 population-based study of 1697 women,^15^ using modified diagnoses, reported that MM was associated with fewer headache days, whereas we found the opposite. Previous clinic-based studies found that menstrual attacks were more severe,^14,16,17,18^ longer in duration,^14,19,20,21,22^ associated with more nausea,^20,23,24^ had greater associated impairment,^20,21^ and were more difficult to treat.^14,19,21,22^ In this study, we found the same associations, but also found that MM was associated with more improvement of migraine attacks during late pregnancy and faster reappearance of migraine attacks post partum. A Norwegian study^25^ of 280 pregnant women reported higher headache intensity during early pregnancy among women with self-reported MM.

### Hormonal Contraceptive–Related vs Spontaneous MM

To our knowledge, there are no previous population-based studies of hormonal contraceptive–related MM, which was specifically called for in the review by Vetvik et al.^7^ We found that women with hormonal contraceptive–related MM had migraine without aura more often than migraine with aura. There were no other significant differences in migraine characteristics between women with spontaneous MM and those with contraceptive-related MM. The *ICHD-3* does not differentiate between the 2 types of menstrual attacks. Because bleeding associated with exogenous hormones disrupts or suppresses the hypothalamic-pituitary-ovarian cycle, the hormonal levels of spontaneous MM and hormonal contraceptive–related MM might not be the same. In a clinical cohort, van Casteren et al^22^ compared characteristics of menstrual days vs nonmenstrual days among users of hormonal contraceptives vs individuals with spontaneous menstruation. Menstrual days among users of hormonal contraceptives were associated with more phonophobia and use of triptans but less nausea. Spontaneous menstrual cycle was associated with increased phonophobia and photophobia and a higher use of analgesics. We could not confirm these results. Thus, women with spontaneous migraine and with hormonal contraceptive–related MM could, for most purposes, be studied together, but should be investigated separately in biochemical studies.

### Proposed Diagnostic Criteria for MM

Recently, a statistical method was proposed to address the issue of diagnostic misclassification of menstrually related migraine in women with CM.^10^ However, the method was too complex to be implemented in clinical practice.^8^ It has been suggested that MM attacks should be defined as *starting* instead of *occurring* on day 1 ± 2 of menstruation.^8^ We used these intervals in our proposed criteria. The proposed definitions solve the following problems with existing criteria. First, rare pure MM is not diagnosed with MM according to the *ICHD-3* diagnostic criteria because the migraine attack frequency is too low to reach 2 of 3 menstrual cycles, but the association between migraine and menstruation is nevertheless absolute. These women are currently excluded and do not belong in any diagnostic criteria because they have rare migraine attacks. To include this important subgroup of women with rare MM attacks, we included them in the diagnostic criteria of pure MM. Second, by chance, 43% women with CM or HFEM will fulfill the current *ICHD-3* diagnostic criteria for MM. We took this into account by excluding CM and HFEM from our proposed diagnostic criteria. Third, in the clinical studies that were used to develop the *ICHD-3* criteria,^17,21,26,27,28^ MM refers to the first day of a migraine attack occurring on or between days –2 to +3 of menstruation, but current *ICHD-3* diagnostic criteria do not clarify that attacks must start within this time frame. We took this into account with our proposed criteria. Fourth, we suggest that the criteria allow for more than 1 attack during the menstrual period.

### Diary-Based vs Self-reported Data

The requirement of a prospective headache and menstruation diary to confirm the diagnosis of MM is still debated.^8^ Recently a clinic-based headache electronic diary study^22^ compared MM and non-MM attacks. Of 3596 patients invited to participate, 500 completed an electronic diary for 1 month and 396 completed the electronic diary for 3 months. The authors^22^ reported that menstrual attacks were associated with longer duration and more severe migraine compared with nonmenstrual attacks. They also found that patients who reported an absolute association with menstruation had 1 or more attacks outside menstruation. To our knowledge, no population-based diary study has obtained 3 months of menstruation-related diary data. Self-report is a common approach both in large-scale epidemiological and genetic studies. In clinical practice, the diagnosis is always based on the patient’s history. Furthermore, prolonged use of a prospective diary leads to a high drop-out rate; thus, in the aforementioned study, only approximately 14% of invited patients were included and only approximately 11% completed a 3-month diary.^22^ In line with *ICHD-3* recommendations (eTable 5 and eAppendix 3 in Supplement 1), our large-scale study did not include diaries. There is always a conflict between data by patients’ history and diary data. In clinical practice, there is no time for diaries before treatment onset. In research studies there is a trade-off between accuracy of a diary and the number and representativity of participants needed. Generally, the *ICHD-3* diagnostic criteria must be applicable at the first patient encounter. Therefore, diaries are not included in our proposed criteria for MM, but they may be useful for accurate patient ascertainment in scientific studies.

### Limitations

This study has limitations that should be addressed. Because our population-based cohort of blood donors had fewer participants with severe comorbidities and fewer participants with 8 or more migraine days per month than the population, we expect that the burden of migraine in women with MM may be even more pronounced. Although the use of patient-reported data made it possible for the study to include a large number of participants with MM, self-reported data involve reporting bias. Prospective diaries are associated with high drop-out rates; thus, given the high numbers needed in this study, our study did not use diaries. Face-to-face interview is ideal but was not possible with high numbers needed for this study. However, the in-cohort validation we performed provided a clear and precise estimate of diagnostic specificity and sensitivity, which were both high. Our suggestions for new diagnostic criteria were not supported by biological data but by logical agreement and a large and validated population-based material. We did not have data on recurrence of migraine attacks, and we also did not have data on the exact day of migraine attack onset and termination. These 2 aspects should be studied in the future.

## Conclusions

In this case-control study of 12 618 Danish individuals with migraine, we found that pure MM and menstrually related migraine are important diagnostic entities. We provided detailed descriptive data and suggested improved diagnostic criteria.

## Notes

Supplement 1. **eAppendix 1.** The Danish Blood Donor Study (DBDS) **eAppendix 2.** Supplemental Code **eFigure.** Flow Diagram of Recruitment **eTable 1.** Current or Previous Use of Prophylactic Treatment **eTable 2.** Pregnancy **eTable 3.** Risk of Diagnostic Misclassification of Menstrually Related Migraine and Pure Menstrual Migraine Calculated by 3 Menstrual Cycles **eTable 4.** Risk of Diagnostic Misclassification of Menstrually Related Migraine and Pure Menstrual Migraine Calculated by 12 Menstrual Cycles **eTable 5.** Chronological Outline of the Definitions of Menstrual Migraine **eAppendix 3.** Historical Development of the Diagnosis of Menstrual Migraine
eReferences

Supplement 2.
Data Sharing Statement
