# Supplementary material for: Population-Based Characterization of Menstrual Migraine and Proposed Diagnostic Criteria
Source: JAMA Netw Open. 2023 May 15;6(5):e2313235. doi: 10.1001/jamanetworkopen.2023.13235 (PMC12549156; doi:10.1001/jamanetworkopen.2023.13235)
Supplement: Supplement 1. — eAppendix 1. The Danish Blood Donor Study (DBDS) eAppendix 2. Supplemental Code eFigure. Flow Diagram of Recruitment eTable 1. Current or Previous Use of Prophylactic Treatment eTable 2. Pregnancy eTable 3. Risk of Diagnostic Misclassification of Menstrually Related Migraine and Pure Menstrual Migraine Calculated by 3 Menstrual Cycles eTable 4. Risk of Diagnostic Misclassification of Menstrually Related Migraine and Pure Menstrual Migraine Calculated by 12 Menstrual Cycles eTable 5. Chronological Outline of the Definitions of Menstrual Migraine eAppendix 3. Historical Development of the Diagnosis of Menstrual Migraine eReferences [file jamanetwopen-e2313235-s001.pdf]

Supplemental Online Content

Chalmer MA, Kogelman LJA, Ullum H, et al. Population-based characterization of menstrual migraine and proposed diagnostic criteria. *JAMA Netw Open*. 2023;6(5):e2313235. doi:10.1001/jamanetworkopen.2023.13235

eAppendix 1. The Danish Blood Donor Study (DBDS)

eAppendix 2. Supplemental Code

eFigure. Flow Diagram of Recruitment

eTable 1. Current or Previous Use of Prophylactic Treatment

eTable 2. Pregnancy

eTable 3. Risk of Diagnostic Misclassification of Menstrually Related Migraine And Pure Menstrual Migraine Calculated by 3 Menstrual Cycles

eTable 4. Risk of Diagnostic Misclassification of Menstrually Related Migraine and Pure Menstrual Migraine Calculated by 12 Menstrual Cycles

eTable 5. Chronological Outline of the Definitions of Menstrual Migraine

eAppendix 3. Historical Development of the Diagnosis of Menstrual Migraine

eReferences

This supplemental material has been provided by the authors to give readers additional information about their work.

© 2023 Chalmer MA et al. *JAMA Network Open.*

eAppendix 1: The Danish Blood Donor Study (DBDS)

The Danish Blood Donor Study (DBDS) started in 2010 and is an ongoing nationwide multicenter, epidemiological cohort and biobank. The demographics of the DBDS has been described in detail elsewhere[1]. We recontacted all participants from the DBDS, who were connected to the Danish public electronic mailing system (e-Boks) between May 2020 and August 2020 (n=127,802). All participants were asked to fill out an extensive migraine questionnaire regardless of whether they were still blood donors. Sex was defined by the unique Danish Civil Registration System number. The diagnostic migraine questionnaire consisted of 105 questions assessing migraine diagnosis, headache frequency, duration, pain characteristics, accompanying symptoms, aura symptoms, autonomic symptoms, allodynia, family history, and treatment response of triptans and over-the-counter simple analgesics (i.e., Paracetamol, Pamol, Panodil, Pinex, Ipren, Ibumax, Ibuprofen, Codimagnyl, Codipar, Acetylsalicylic Acid, and Treo). Acute treatment effect was scaled from 0-10, and efficacy was defined as the interval from 50% pain relief to pain freedom, i.e., the standard effect measurement in clinical trials. In total 62,672 participants answered the questionnaire and entered our case-control study with the primary aim to study sex differences in the presentation of migraine. Diagnosis of migraine was made by applying the criteria of the ICHD-3 (MAC and JO). Individuals with missing data regarding migraine characteristics, not allowing for assessment of a migraine diagnosis, were excluded from the main analysis, and set as controls. Participants who fulfilled a migraine diagnosis (N=12,618) constituted the Danish Migraine Population Cohort (DaMP)[2]. For parameters relevant to the present study such as self-reported, health-related quality of life and socio-economic factors, the DaMP cohort was representative of the general Danish population, however, there were fewer participants with severe comorbidities[2–4]. The questionnaire was in-cohort validated using a validated semi-structured telephone interview[5,6] performed by a specially trained neurology resident (MAC) in 500 randomly selected responders. The semi-structured interview assessed migraine with aura (MA) and migraine without aura (MO) separately in detail, including frequency, duration, pain, aura, accompanying- and autonomic symptoms. The overall migraine diagnosis, i.e., all migraine, had a specificity of 93% and a sensitivity of 93%, giving a positive predictive value of

© 2023 Chalmer MA et al. *JAMA Network Open.*

97%. Blood donors enter a quarantine period if they use analgesics of any form, thus, the risk of an overestimation of medication overuse among blood donors is very small.

eAppendix 2: Supplemental Code:

```
options(tidyverse.quiet = TRUE)
options(dplyr.summarise.inform = FALSE)

library(tidyverse)
```

© 2023 Chalmer MA et al. *JAMA Network Open.*

```
SMM <- function(dm=5,period_length=28,n_periods=3,attacks=3,migraineurs=10000) {
  #####  Parameter definition ####
  ## dm = days with menstruation
  ## period_length = menstruation period length in days, i.e. day 0 to the next day 0
  ## n_periods = Periods to be simulated
  ## attacks = Total numbers of attacks during the simulation period
  ## migraineurs = Number of patients popultion simulated

  ##### Generate menstruation Diary
  period <- map_dfr(seq_len(n_periods),
                    ~ (tibble(day=1:period_length) %>%
                         mutate(menstruation=if_else(day<=dm,"YES","NO"))
                    )
  ) %>%
    mutate(attack_day = row_number())

  ### generate simulation dataset
  migs_table  <-
    paste("attack",1:attacks,sep="_") %>%
    rlang::rep_named(list(logical())) %>%
    as_tibble()

  for (rows in 1:migraineurs) {
    migs_table(rows,) <- as.list(replicate(attacks,sample(1:(period_length*n_periods),1,rep=F)))
  }

  migs_table <-
    migs_table %>%
    mutate(ID=row_number()) %>%
    gather(attack_n,attack_day,-ID) %>%
    rowwise() %>%
    left_join(period %>%  ### Merge simulation dataset with menstruation diary
                select(attack_day,menstruation),
              by = "attack_day")

  ### count incidences with MM
  out <- migs_table %>%
    group_by(ID,menstruation) %>%
    filter(menstruation=="YES") %>%
    summarise(MM_attacks=n()) %>%
    ungroup() %>%
    group_by(MM_attacks) %>%
    summarise(Patients=n())

  return(out)

}
```

© 2023 Chalmer MA et al. *JAMA Network Open.*

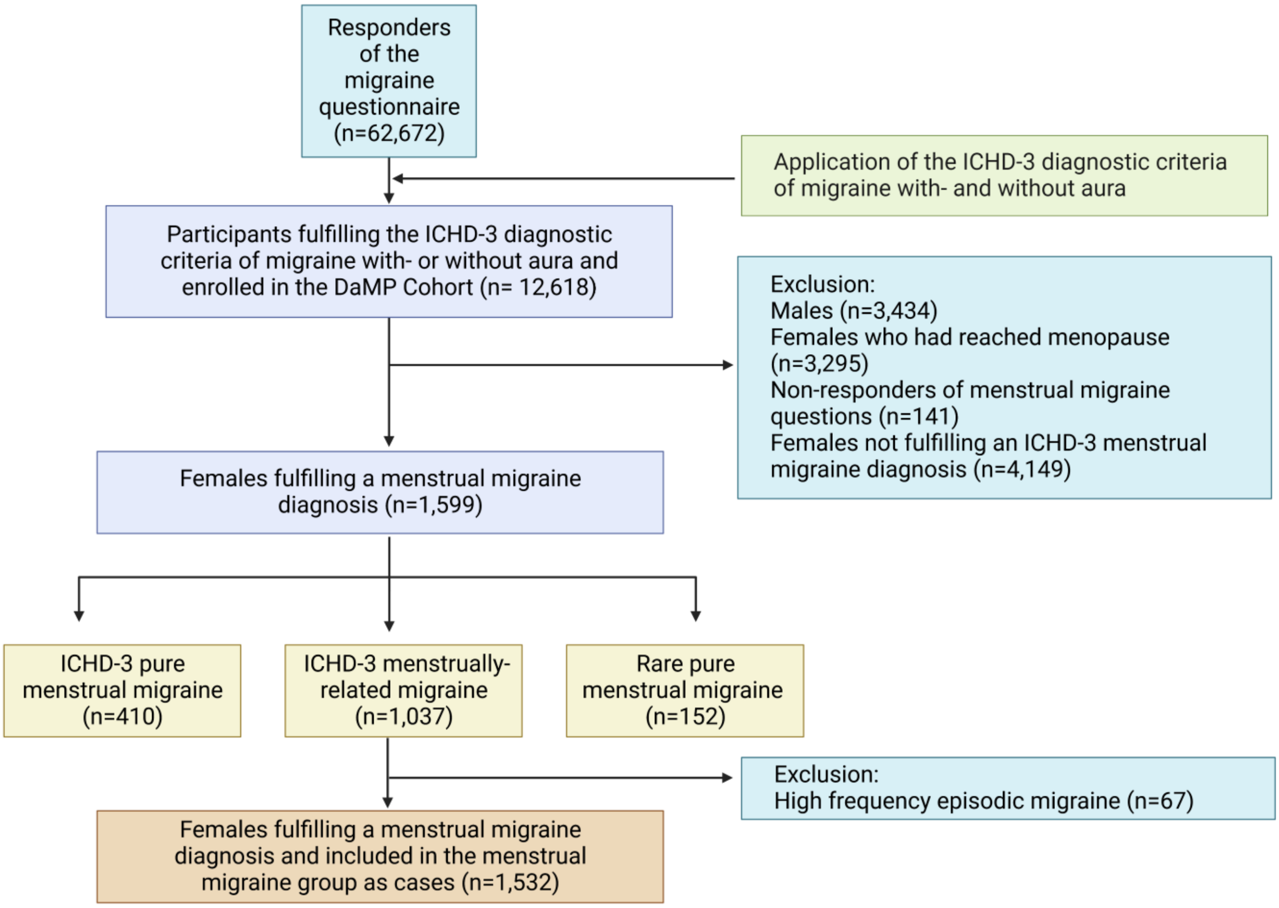

eFigure 1: Flow diagram of recruitment. DBDS=Danish Blood Donor Study. ICHD-3=International Classification of Headache Disorders third edition. DaMP=Danish Migraine Population

© 2023 Chalmer MA et al. *JAMA Network Open.*

eTable 1: Current or previous use of prophylactic treatment				
	**Menstrual migraine (N = 1,532)**	**Non-menstrual migraine (N = 4,216)**		
**Variables**	**N (%)**	**N (%)**	**OR (95%CI)**	***P* Value**
**Migraine prophylactic treatment**				
**Angiotensin-converting enzyme**	2 (0.1)	12 (0.3)	0.45 (0.07-1.67)	0.30
**Angiotensin II receptor blocker**	13 (0.8)	35 (0.8)	0.99 (0.50-1.84)	0.98
**Beta blockers**	40 (2.6)	96 (2.3)	1.13 (0.77-1.63)	0.52
**Calcium channel blockers**	7 (0.5)	6 (0.1)	3.04 (1.00-9.48)	<.001
**Antidepressants**	12 (0.8)	22 (0.5)	1.51 (0.72-3.01)	0.25
**Anticonvulsants**	9 (0.6)	26 (0.6)	0.93 (0.41-1.93)	0.86
**Hormone therapy**	17 (1.1)	9 (0.2)	5.09 (2.31-12.00)	<.001
**Botulinum toxin**	14 (0.1)	20 (0.5)	1.89 (0.93-3.73)	<.001
**Calcitonin gene-related peptide therapy**	0 (0)	6 (0.1)	0.00	0.95

eTable 1: Current or previous use of migraine prophylactic treatment. Non-menstrual migraine was used as reference (ref) in the adjusted logistic regression analysis. Results are presented as numbers (n), percentages (%) and odds ratios (OR) with corresponding 95% confidence intervals (CI).

© 2023 Chalmer MA et al. *JAMA Network Open.*

eTable 2: Pregnancy				
	**Menstrual migraine (N = 1,021)**	**Non-menstrual migraine (N = 2,485)**		
**Variables**	**N (%)**	**N (%)**	**OR (95%CI)**	***P*Value**
**Status of migraine attack frequency during 2nd and 3rd trimester compared with prepartum. Migraine attacks were:**				
**Very frequent**	6 (0.6)	36 (1.4)	Ref	NA
**Frequent**	38 (3.7)	107 (4.3)	2.10 (0.87-5.89)	0.12
**Unchanged**	40 (3.9)	129 (5.2)	1.74 (0.73-4.87)	0.25
**Rare**	79 (7.8)	91 (3.7)	5.10 (2.17-14.0)	<.001
**Very rare**	78 (7.7)	135 (5.4)	3.36 (1.44-9.19)	<.001
**Disappeared**	146 (14.3)	163 (6.6)	5.25 (2.30-14.2)	<.001
**Status of migraine attacks postpartum compared with 2nd and 3rd trimester. Migraine attacks were:**				
**Very frequent**	27 (2.6)	33 (1.3)	Ref	NA
**Frequent**	139 (13.7)	201 (8.1)	0.87 (0.50-1.51)	0.61
**Unchanged**	101 (9.9)	202 (8.1)	0.61 (0.35-1.08)	0.09
**Rare**	77 (7.6)	144 (5.8)	0.66 (0.37-1.18)	0.16
**Very rare**	77 (7.6)	146 (5.9)	0.65 (0.36-1.16)	0.14
**Disappeared**	40 (3.9)	109 (4.9)	0.46 (0.24-0.85)	0.01
**Return of migraine attacks postpartum:**				
**Less than a week**	14 (1.5)	34 (1.5)	1.81 (1.49-2.21)	0.07
**One week**	9 (0.9)	20 (0.9)	2.11 (0.90-4.60)	0.07
**One month**	110 (11.5)	156 (6.8)	3.19 (2.40-4.25)	<.001
**Six months**	292 (30.6)	427 (18.6)	3.04 (2.47-3.75)	<.001
**One year or longer**	290 (30.4)	668 (29.1)	1.81 (1.49-2.21)	<.001
**There were no migraines**	239 (25.1)	990 (43.1)	Ref	NA

eTable 2: Pregnancy in females with menstrual and non-menstrual migraine. Females with non-menstrual migraine who had given birth, were used as reference (ref) in the adjusted logistic regression analysis (adjusted for age). Results are presented as numbers (n), percentages (%) and odds ratios (OR) with corresponding 95% confidence intervals (CI). First pregnancy, if the female had more than one total pregnancy. Only females who had given birth were included in this analysis.

© 2023 Chalmer MA et al. *JAMA Network Open.*

eTable 3: Risk of diagnostic misclassification of menstrually related migraine and pure menstrual migraine calculated by three menstrual cycles

	total_attacks	mm_related	frq.x	mm_pure	frq.y	mm_relatedOLD	frq.x.x	mm_pureOLD	frq.y.y
1	1	17853	17.85	17728	17.73	NA	NA	NA	NA
2	2	32709	32.71	3258	3.26	3255	3.26	3109	3.11
3	3	8252	8.25	557	0.56	8442	8.44	592	0.59
4	4	14890	14.89	95	0.10	14891	14.89	97	0.10
5	5	4274	4.27	24	0.02	21934	21.93	15	0.01
6	6	7428	7.43	5	0.00	29204	29.20	2	0.00
7	7	2251	2.25	0	0.00	36397	36.40	0	0.00
8	8	3829	3.83	0	0.00	43537	43.54	0	0.00
9	9	1165	1.17	0	0.00	49358	49.36	0	0.00
10	10	2050	2.05	0	0.00	55798	55.80	0	0.00
11	11	699	0.70	0	0.00	60780	60.78	0	0.00
12	12	1135	1.14	0	0.00	65995	66.00	0	0.00
13	13	347	0.35	0	0.00	70431	70.43	0	0.00
14	14	556	0.56	0	0.00	74175	74.17	0	0.00
15	15	182	0.18	0	0.00	77628	77.63	0	0.00
16	16	311	0.31	NA	NA	80718	80.72	NA	NA
17	17	103	0.10	NA	NA	83261	83.26	NA	NA
18	18	178	0.18	NA	NA	85723	85.72	NA	NA
19	19	63	0.06	NA	NA	87897	87.90	NA	NA
20	20	104	0.10	NA	NA	89613	89.61	NA	NA
21	21	37	0.04	NA	NA	91003	91.00	NA	NA
22	22	66	0.07	NA	NA	92402	92.40	NA	NA
23	23	31	0.03	NA	NA	93351	93.35	NA	NA
24	24	28	0.03	NA	NA	94501	94.50	NA	NA
25	25	15	0.01	NA	NA	95201	95.20	NA	NA
26	26	17	0.02	NA	NA	96014	96.01	NA	NA
27	27	6	0.01	NA	NA	96657	96.66	NA	NA
28	28	16	0.02	NA	NA	97161	97.16	NA	NA
29	29	4	0.00	NA	NA	97573	97.57	NA	NA
30	30	6	0.01	NA	NA	97885	97.89	NA	NA

© 2023 Chalmer MA et al. *JAMA Network Open.*

eTable 4: Risk of diagnostic misclassification of menstrually related migraine and pure menstrual migraine calculated by 12 menstrual cycles

	total_attacks	mm_related	frq.x	mm_pure	frq.y	mm_relatedOLD	frq.x.x	mm_pureOLD	frq.y.y
1	1	17818	17.82	17775	17.77	NA	NA	NA	NA
2	2	32571	32.57	3162	3.16	3214	3.21	3246	3.25
3	3	8433	8.43	622	0.62	8494	8.49	537	0.54
4	4	14951	14.95	122	0.12	14838	14.84	98	0.10
5	5	4112	4.11	15	0.01	21921	21.92	15	0.01
6	6	7470	7.47	6	0.01	29045	29.05	1	0.00
7	7	2251	2.25	0	0.00	36412	36.41	0	0.00
8	8	3832	3.83	0	0.00	43489	43.49	0	0.00
9	9	1236	1.24	0	0.00	49824	49.82	0	0.00
10	10	2094	2.09	0	0.00	55705	55.70	0	0.00
11	11	682	0.68	0	0.00	61227	61.23	0	0.00
12	12	1113	1.11	0	0.00	66113	66.11	0	0.00
13	13	356	0.36	0	0.00	70071	70.07	0	0.00
14	14	590	0.59	0	0.00	74110	74.11	0	0.00
15	15	217	0.22	0	0.00	77748	77.75	0	0.00
16	16	331	0.33	0	0.00	80664	80.66	0	0.00
17	17	123	0.12	0	0.00	83312	83.31	0	0.00
18	18	186	0.19	0	0.00	85845	85.84	0	0.00
19	19	59	0.06	0	0.00	87890	87.89	0	0.00
20	20	113	0.11	0	0.00	89698	89.70	0	0.00
21	21	44	0.04	0	0.00	90934	90.93	0	0.00
22	22	56	0.06	0	0.00	92287	92.29	0	0.00
23	23	25	0.03	0	0.00	93550	93.55	0	0.00
24	24	27	0.03	0	0.00	94422	94.42	0	0.00
25	25	7	0.01	0	0.00	95329	95.33	0	0.00
26	26	34	0.03	0	0.00	96013	96.01	0	0.00
27	27	6	0.01	0	0.00	96626	96.63	0	0.00
28	28	12	0.01	0	0.00	97185	97.18	0	0.00
29	29	2	0.00	0	0.00	97615	97.61	0	0.00
30	30	4	0.00	0	0.00	97943	97.94	0	0.00

© 2023 Chalmer MA et al. *JAMA Network Open.*

eTable 5: Chronological outline of the definitions of menstrual migraine		
**Year**	**Author**	**Definition**
1983	*D'Allesandro*[7]	Migraine only or predominately during menses
1984	*Solbach*[8]	Any migraine headache which occurs 3 days prior to the menstrual flow, during the time of the flow, or three days following.
1987	*Digre*[9]	Common migraine occuring during the week before or the week of menstruation; the woman is headache free for the remainder of the cycle.
1988	*ICHD-I*[10]	The term menstrual migraine was introduced, but no accepted criteria for menstrual migraine were given, due to lack of epidemiological studies
1990	*MacGregor*[11]	Migraine attacks which occur regularly on or between days -2 to +3 of the menstrual cycle and at no other time.
1991	*Nattero*[12]	The experience of migraine attacks exclusively prior to, during or at the end of menstruation (i.e., true menstrual migraine).
1996	*MacGregor*[13]	Migraine attacks occurring within day 1±2 days of menstruation (i.e., on or between two days prior to menstruation and the first two days of menstruation) and at no other time of the cycle.
2003	*ICHD-2*[14]	*A1.1.1 Pure menstrual migraine without aura:* A. Attacks, in a menstruating woman, fulfilling criteria for 1.1 Migraine without aura. B: Attacks occur exclusively on day 1 ± 2 (ie, days -2 to +3) of menstruation in at least two out of three menstrual cycles and at no other times of the cycle.<br><br>*A1.1.2 Menstrually-related migraine without aura:* A. Attacks, in a menstruating woman, fulfilling criteria for 1.1 Migraine without aura. B: Attacks occur on day 1±2 (*ie*, days -2 to +3) of menstruation in at least two out of three menstrual cycles and additionally at other times of the cycle.<br><br>*Documented, prospectively recorded evidence, kept for a minimum of three cycles, is necessary to confirm the diagnosis as many women over-report an association between attacks and menstruation.*
2010	*Marcus*[15]	*A probability model menstrual migraine (Probability menstrual migraine:* Migraine episodes with pain severity ≥4 occurring with a greater probability ($P < .05$) on menstrual days (-2 to +3) compared with non-menstrual days.

© 2023 Chalmer MA et al. *JAMA Network Open.*

eTable 5 continued: Chronological outline of the definitions of menstrual migraine		
**Year**	**Author**	**Definition**
2018	*ICHD-3*[16]	*A1.1.1 Pure menstrual migraine without aura:* A: Attacks, in a menstruating woman, fulfilling criteria for 1.1 Migraine without aura and criterion B below. B: Occurring exclusively on day 1±2 (i.e. days -2 to +3) of menstruation in at least two out of three menstrual cycles and at no other times of the cycle.<br><br>*A1.1.2 Menstrually related migraine without aura:* A: Attacks, in a menstruating woman,1 fulfilling criteria for 1.1 Migraine without aura and criterion B below. B: Occurring on day 1±2 (i.e. days -2 to +3) of menstruation in at least two out of three menstrual cycles, and additionally at other times of the cycle.<br><br>*A1.2.0.1 Pure menstrual migraine with aura:* A: Attacks, in a menstruating woman, fulfilling criteria for 1.2 Migraine with aura and criterion B below. B: Occurring exclusively on day 1±2 (i.e. days -2 to +3) of menstruation in at least two out of three menstrual cycles and at no other times of the cycle.<br><br>*A1.2.0.2 Menstrually related migraine with aura:* A: Attacks, in a menstruating woman, fulfilling criteria for 1.2 Migraine without aura and criterion B below. B: Occurring on day 1±2 (i.e. days -2 to +3) of menstruation in at least two out of three menstrual cycles, and additionally at other times of the cycle.<br><br>*For research purposes a prospective diary is recommended, but this is not mandatory for clinical diagnosis.*
2019	*Barra*[17]	*Statistical Menstrual Migraine (α):* 1) Migraine without aura. 2) A trimmed (migraine-locked free) headache diary's one-sided Fischer Exact mid-p corrected p -value < α on a test of $\Delta\mu = 0$.
2022	*Verhagen*[18]	*Proposal for menstrual migraine diagnostic criteria*: A. Attacks, in a menstruating woman, fulfilling criteria for migraine with or without aura. B. Starting on day 1±2 (i.e., days -2 to +3) of menstruation in at least two out of three menstrual cycles (note: attacks may occur additionally at other times of the cycle).<br><br>*A prospective (E-)diary is strongly recommended for both research purposes and clinical practice.*

eTable 5: Chronological outline of the definitions of menstrual migraine. Definitions and diagnostic criteria are adapted from the respective articles which are referred in the table.

eAppendix 3: Historical Development of the Diagnosis of Menstrual Migraine

“Menstrual migraine” has been recognized since the time of Hippocrates as >>*agitated blood seeking a way of escape*<<[19]. For decades, migraine was reported as part of the premenstrual syndrome[20] and the term “menstrual migraine” was lacking a precise definition[13]. The term was frequently used, but, there were different interpretations of the diagnostic criteria, which resulted in large variation in the reported prevalences of menstrual migraine[21–29]. In its first edition in 1988, the International Classification of Headache Disorders (ICHD) introduced the term menstrual migraine[10], but, due to lack of epidemiological studies at this point, generally accepted criteria for the entity were not proposed, and it was concluded that further studies were necessary. This led to more studies on menstrual migraine, and in 1990 MacGregor introduced the first proposal of a definition of menstrual migraine[11], and we have highlighted all previous proposed diagnostic criteria for menstrual migraine in eTable 5. The first explicit criteria for menstrual migraine were published with the ICHD-2[14] and consisted of: A1.1.1 Pure menstrual migraine without aura and A1.1.2 Menstrually-related migraine without aura. The criteria only included a diagnosis of menstrual migraine without aura, because perimenstrual attacks were typically reported as migraine without aura[30]. It was required that migraine must occur in two of three consecutive menstruations. In the ICHD-3[16], the criteria remained the same but menstrual migraine in relation with migraine with aura was also allowed to encourage better characterization of menstrual migraine. Prospective headache diary was not mandatory for clinical diagnosis but was encouraged for research purposes to avoid overreporting. The requirement of diaries for the diagnosis of menstrual migraine is still debated[15,18,31], and menstrual migraine diagnoses remain in the appendix because males cannot have menstrual migraine.

© 2023 Chalmer MA et al. *JAMA Network Open.*

eReferences

1. Erikstrup C, Sørensen E, Nielsen KR, et al. Cohort Profile: The Danish Blood Donor Study. *Int J Epidemiol*. Epub ahead of print 4 October 2022. DOI: 10.1093/ije/dyac194.

2. Chalmer MA, Kogelman LJA, Callesen I, et al. Sex differences in clinical characteristics of migraine and its burden: A population-based study. *Eur J Neurol*. Epub ahead of print 10 March 2023. DOI: 10.1111/ene.15778.

3. Rigas AS, Skytthe A, Erikstrup C, et al. The healthy donor effect impacts self-reported physical and mental health – results from the Danish Blood Donor Study (DBDS). *Transfus Med* 2019; 29: 65–69.

4. Burgdorf KS, Simonsen J, Sundby A, et al. Socio-demographic characteristics of Danish blood donors. *PLoS One* 2017; 12: 1–11.

5. Rasmussen BK, Jensen R, Olesen J. Questionnaire Versus Clinical Interview in the Diagnosis of Headache. *Headache J Head Face Pain* 1991; 31: 290–295.

6. Gervil M, Ulrich V, Olesen J, et al. Screening for migraine in the general population: validation of a simple questionnaire. *Cephalalgia* 1998; 18: 342–348.

7. D'alessandro R, Gamberini G, Lozito A, et al. Menstrual migraine: intermittent prophylaxis with a timed-release pharmacological formulation of dihydroergotamine. *Cephalalgia* 1983; 3: 156–158.

8. Solbach P, Sargent J, Coyne L. Menstrual Migraine Headache: Results of a Controlled, Experimental, Outcome Study of Non-drug Treatments. *Headache J Head Face Pain* 1984; 24: 75–78.

9. Digre K, Damasio H. Menstrual Migraine: Differential Diagnosis, Evaluation, and Treatment. *Clin Obstet Gynecol* 1987; 30: 417–430.

10. Headache Classification Committee of the International Headache Society. Classification and diagnostic criteria for headache disorders, cranial neuralgias and facial pain. Headache Classification Committee of the International Headache Society. *Cephalalgia* 1988; 8: 1–96.

11. MacGregor EA, Chia H, Vohrah RC, et al. Migraine and menstruation: A pilot study. *Cephalalgia* 1990; 10: 305–310.

12. Nattero G, Allais G, De Lorenzo C, et al. Biological and clinical effects of naproxen sodium in patients with menstrual migraine. *Cephalalgia* 1991; 11: 201–202.

13. MacGregor E. "Menstrual" Migraine: Towards a Definition. *Cephalalgia* 1996; 16: 11–21.

14. Headache Classification Committee of the International Headache Society. The International Classification of Headache Disorders (second edition). *Cephalalgia* 2004; 24: 1–160.

15. Marcus DA, Bernstein CD, Sullivan EA, et al. A prospective comparison between ICHD-II and probability menstrual migraine diagnostic criteria. *Headache* 2010; 50: 539–550.

16. Headache Classification Committee of the International Headache Society. The International Classification of Headache Disorders, 3rd edition. *Cephalalgia* 2018; 38: 1–211.

17. Barra M, Dahl FA, MacGregor EA, et al. Identifying menstrual migraine- improving the diagnostic criteria using a statistical method. *J Headache Pain* 2019; 20: 1–12.

18. Verhagen IE, Spaink HA, van der Arend BW, et al. Validation of diagnostic ICHD-3 criteria for menstrual migraine. *Cephalalgia* 2022; 033310242210990.

19. DALTON K. PROGESTERONE SUPPOSITORIES AND PESSARIES IN THE TREATMENT OF MENSTRUAL MIGRAINE. *Headache J Head Face Pain* 1973; 12: 151–159.

© 2023 Chalmer MA et al. *JAMA Network Open.*

20. Greene R, Dalton K. The Premenstrual Syndrome. *BMJ* 1953; 1: 1007–1014.

21. Russell M, Rasmussen B, Fenger K, et al. Migraine Without Aura and Migraine with Aura Are Distinct Clinical Entities: A Study of Four Hundred and Eighty-Four Male and Female Migraineurs From the General Population. *Cephalalgia* 1996; 16: 239–245.

22. Karlı N, Baykan B, Ertaş M, et al. Impact of sex hormonal changes on tension-type headache and migraine: A cross-sectional population-based survey in 2,600 women. *J Headache Pain* 2012; 13: 557–565.

23. Dzoljic E, Sipetic S, Vlajinac H, et al. Prevalence of menstrually related migraine and nonmigraine primary headache in female students of Belgrade University. *Headache* 2002; 42: 185–193.

24. Cupini LM, Matteis M, Calabresi P, et al. Sex-Hormone-Related events in migrainous females. A clinical comparative study between migraine with aura and migraine without aura. *Cephalalgia* 1995; 15: 140–144.

25. Granella F, Sances G, Pucci E, et al. Migraine with aura and reproductive life events: A case control study. *Cephalalgia* 2000; 20: 701–707.

26. Tepper SJ, Zatochill M, Szeto M, et al. Development of a simple menstrual migraine screening tool for obstetric and gynecology clinics: The menstrual migraine assessment tool. *Headache* 2008; 48: 1419–1425.

27. Couturier EGM, Bomhof MAM, Neven AK, et al. Menstrual migraine in a representative Dutch population sample: Prevalence, disability and treatment. *Cephalalgia* 2003; 23: 302–308.

28. Mattsson P. Hormonal factors in migraine: A population-based study of women aged 40 to 74 years. *Headache* 2003; 43: 27–35.

29. Vetvik KG, MacGregor EA, Lundqvist C, et al. Prevalence of menstrual migraine: A population-based study. *Cephalalgia* 2014; 34: 280–288.

30. MacGregor EA. Classification of Perimenstrual Headache: Clinical Relevance. *Curr Pain Headache Rep* 2012; 16: 452–460.

31. Vetvik KG, MacGregor EA. Menstrual migraine: a distinct disorder needing greater recognition. *Lancet Neurol* 2021; 20: 304–315.

© 2023 Chalmer MA et al. *JAMA Network Open.*
