# Supplementary material for: Population-Based Characterization of Menstrual Migraine and Proposed Diagnostic Criteria
Source: JAMA Netw Open. 2023 May 15;6(5):e2313235. doi: 10.1001/jamanetworkopen.2023.13235 (PMC12549156; doi:10.1001/jamanetworkopen.2023.13235)
Supplement: Supplement 2. — Data Sharing Statement [file jamanetwopen-e2313235-s002.pdf]

Data Sharing Statement

Chalmer. Population-Based Characterization of Menstrual Migraine and Proposed Diagnostic Criteria. *JAMA Netw Open*. Published May 15, 2023.
doi:10.1001/jamanetworkopen.2023.13235

Data
Data available: No

Additional Information
Explanation for why data not available: Data are available from the corresponding authors upon reasonable request and require both a material transfer agreement and memorandum of understanding in order to obtain ethical and data protection agency approval.
